# Efficacy and Tolerability of Pridinol Mesylate Versus Quinine Sulfate in the Treatment of Nocturnal Leg Cramps: A Propensity Score-Matched Real-World Analysis of Depersonalized 4-Week Data from the German Pain e-Registry (PRISCILA Study)

**DOI:** 10.3390/jcm15051708

**Published:** 2026-02-24

**Authors:** Michael A. Überall, Herbert Schreiber

**Affiliations:** 1 IFNAP–Private Institute of Neurological Sciences, O. Meany-MDPM GmbH, Nordostpark 51, 90411 Nürnberg, Germany; 2 Neurological Group Practice, Pfauengasse 8, 89073 Ulm, Germany

**Keywords:** nocturnal leg cramps, quinine sulfate, pridinol, antispasmodics, real-world evidence, German Pain e-Registry

## Abstract

**Background:** Nocturnal leg cramps (NLCs) are common, especially in older adults, and may cause substantial distress, sleep disturbance, and functional impairment. Despite widespread clinical use of quinine sulfate (QUI), safety concerns limit its use. Pridinol mesylate (PRI), a centrally acting antispasmodic, may offer a promising alternative in clinical practice. **Objective:** To evaluate the clinical effectiveness and tolerability of PRI versus QUI in patients with NLCs. **Methods:** We conducted a retrospective, non-interventional, propensity score-matched analysis of anonymized routine data from 1722 adult patients (861 per group) with NLCs from the German Pain e-Registry (GPeR). Patients initiating either PRI or QUI between 2018 and 2023 were included. The primary outcome was a predefined composite responder rate (≥50% reduction in NLC frequency, duration, and affected nights, with no treatment discontinuation due to adverse drug reactions [ADRs] or inefficacy). Secondary outcomes included pain intensity, quality-of-life, disability, and ADR frequency. **Results:** PRI treatment resulted in a significantly higher responder rate (56.9%) compared to QUI (48.4%, *p* < 0.001; NNT = 12) due to greater short-term reductions in NLC episodes, duration, and pain intensity. The overall ADR rates were numerically higher with PRI (8.6%) than with QUI (6.7%), but discontinuation rates due to ADRs or inefficacy were comparable between groups and occurred in 3.1/2.6% with PRI/QUI (*p* = 0.865). **Conclusions:** In this large, real-world, propensity score-matched analysis, pridinol treatment was associated with a modest short-term advantage over quinine in several efficacy outcomes, while overall tolerability appeared broadly comparable. Given the retrospective, non-interventional design and the limited 4-week observation period, these findings should be interpreted as hypothesis-generating rather than confirmatory.

## 1. Introduction/Background

Nocturnal leg cramps (NLCs) are sudden, painful, involuntary muscle contractions typically affecting the calf, thigh, or foot during rest, predominantly at night [1,2,3,4]. Prevalence increases with age, with studies reporting occurrences in up to 56% of older adults and in approximately 6% of children [5,6,7,8]. Despite their frequency and their substantial impact on sleep, everyday function, and quality-of-life, NLCs are underreported and often inadequately treated [7,9,10].

Patients suffering from NLCs frequently describe intense, sleep-disrupting pain that leads to daytime fatigue, reduced activity, and psychological burden [4,7]. In population-based studies, over 40% of older adults reported NLC episodes occurring more than three times per week, with up to 21% describing symptoms as “very distressing” [8,9,11]. This burden is magnified in vulnerable populations such as the elderly, those with metabolic disturbances, or during pregnancy [1,12,13].

Despite the significant clinical burden, treatment options for NLC remain limited. Non-pharmacologic interventions (hydration, stretching) are commonly recommended but often insufficient. Pharmacologically, quinine sulfate (QUI) remains the most frequently prescribed agent and is currently the only substance with a positive recommendation in the German Neurological Association guidelines (DGN-S1) for the second-line pharmacological treatment of NLCs [14].

Quinine exerts its antispasmodic effect through a dual peripheral mechanism. At the neuromuscular junction, it acts as a functional non-competitive inhibitor of nicotinic acetylcholine receptors (nAChRs), producing both neurotropic effects—by blocking voltage-gated ion channels leading to reduced end-plate potentials and thus impairing signal transmission from nerve to muscle—and myotropic effects, thereby reducing local muscle excitability [15]. This mechanism renders QUI effective regardless of the underlying etiology of the muscle cramps. Furthermore, due to its purely peripheral mode of action, QUI does not cause central nervous system side effects such as sedation or dependence, nor does it impair the ability to drive or operate machinery—advantages of high practical relevance in everyday life. Nevertheless, its use is constrained by the potential occurrence of rare but serious adverse drug reactions (ADRs), including hemolytic uremic syndrome, disseminated intravascular coagulation, thrombocytopenia, cardiac arrhythmias, and hypersensitivity reactions [16,17], which have led to significant regulatory restrictions in several countries, including the United States, Australia, and New Zealand [18,19,20].

Pridinol mesylate (PRI) is a centrally acting, non-benzodiazepine, antispasmodic agent approved in Germany, the UK, Poland, and Italy for the treatment of painful muscle spasms and nocturnal leg cramps [21]. Its pharmacological mechanism involves antagonistic effects on central muscarinic acetylcholine receptors, modulating spinal and supraspinal reflex activity to alpha-motoneurons and thereby reducing pathological muscle hyperactivity [22]. Although observational data from real-world registries and recent meta-analyses suggest clinically meaningful benefits of PRI in the management of musculoskeletal pain and muscle cramping—particularly among elderly patients and in comparison to NSAIDs [23,24,25,26]—PRI has not yet been incorporated into current treatment guidelines for nocturnal leg cramps, including the German DGN-S1 guidelines [14]. The primary reason for this omission lies in the absence of randomized, placebo-controlled clinical trials, which remain the gold standard for guideline-level recommendations. Even though its mode of action may raise concerns regarding anticholinergic side effects, PRI has been classified as a score of 1 according to the CRIDECO Anticholinergic Load Scale, indicating a low central anticholinergic risk—a relevant consideration in clinical decision-making, particularly for multimorbid and geriatric patients [27].

To our knowledge, no head-to-head comparative real-world study between QUI and PRI for NLCs has yet been either published or performed. The present study (PRISCILA) addresses this gap by using large, matched, retrospective cohorts from the German Pain e-Registry (GPeR) to compare the clinical effectiveness and tolerability of these two agents for NLC in routine care.

## 2. Study Objective

Given the limited pharmacological options for the treatment of NLCs and the ongoing debate surrounding the use of QUI, this study was designed to provide real-world evidence by comparing the effectiveness and tolerability of PRI and QUI under routine outpatient care conditions. Hence, the primary objective of this study was evaluation of 4-week responses in comparable patient populations of the GPeR with insufficient symptom relief in response to self-medication and/or non-pharmacological countermeasures for NLC, who received a prescription for QUI or alternatively PRI.

Secondary objectives focused on the overall prevalence and severity of ADRs and ADR- and inefficiency-related treatment discontinuations in both study cohorts.

## 3. Methods

### 3.1. Study Design and Setting

PRISCILA (EU PAS registration number: 1000000105) is a retrospective, non-interventional, open-label, post-marketing observational study using anonymized data collected prospectively in the GPeR, a nationwide documentation system for routine care in pain medicine. The data were mirrored from patients treated between 1 January 2018 and 30 September 2023.

### 3.2. Inclusion and Exclusion Criteria

There was no formal sample size calculation for this retrospective real-world analysis. Instead, all eligible patient datasets documented in the GPeR between 1 January 2018 and 30 September 2023 were screened for inclusion. Eligible datasets were those of patients with a physician-confirmed diagnosis of NLCs who had newly initiated treatment with either QUI or PRI during the specified observation period. Treatment initiation was defined as the first intake of the respective index medication following a minimum of 12 weeks without prior exposure to the same agent. The date of the first dose was designated as the index date and marked the beginning of the predefined 4-week evaluation period.

All treatments assessed in this analysis were initiated as part of routine clinical care, based solely on individual patient needs and shared decision-making between physician and patient, without external influence or protocol-driven criteria. Reasons for initiating index therapy included, among others, a decline in everyday functional capacity, increased NLC frequency or intensity, or prior nonresponse or intolerance to non-pharmacological or pharmacological measures.

To be included in the analysis, patients had to be at least 18 years of age at the time of treatment initiation, exhibit a documented diagnosis of NLCs based on ICD-10 coding (R25.2), and have initiated treatment with either PRI or QUI for the first time during the study period. In addition, they were required to have complete and plausible documentation for all relevant baseline variables and for the entire 4-week observation window. Importantly, patients who did not maintain treatment for the entire four weeks—for example due to ADRs or a lack of efficacy—were not excluded. Instead, the last intake date and the reason for discontinuation were recorded and these patients were retained in the analysis following a modified intention-to-treat (mITT) approach.

Data were excluded from the study if patients had an active malignancy or were undergoing cancer therapy at the time of data collection, had a history of spinal surgery (which could independently affect neuromuscular cramp activity) or were concurrently receiving other centrally acting analgesics or antispasmodics during the observation period, which could confound the evaluation of treatment effects. Additionally, datasets were excluded if critical baseline or follow-up data were missing or implausible, thereby precluding valid endpoint analysis.

### 3.3. Treatment Exposure

Therapy with PRI or QUI was prescribed based on individual physician–patient decisions and followed approved dosing recommendations. Treatment duration was defined from first intake to final reported use within the 28-day observation window. Treatment exposure was characterized by treatment duration, cumulative dose, number of tablets taken, and defined daily doses (DDDs) during the predefined 28-day observation period.

### 3.4. Propensity Score Matching and Cohort Construction

To minimize baseline confounding factors and ensure the comparability of treatment groups, a propensity score matching (PSM) approach was applied. All eligible patient datasets were first stratified by treatment assignment into two cohorts: cohort A, comprising patients treated with QUI, and cohort B, comprising patients treated with PRI.

A logistic regression model was developed to estimate each patient’s probability of receiving either PRI or QUI, conditional on predefined key baseline characteristics [28]. The propensity score was defined as the predicted probability of being assigned to one of the two treatment groups based on the fitted model. The covariates included in the propensity score model were selected a priori based on clinical relevance and empirical association with treatment assignment. Specifically, the model incorporated the following variables: age, sex, disease duration, number of NLCs per night, cumulative number of NLCs per week, NLC cramp duration, and NLC pain intensity (0–100 mm VAS).

Patients in the two cohorts were then matched using nearest neighbor matching without replacement (NNWOR), with a caliper width of 0.15 standard deviations of the logit of the propensity score, to avoid poor matches and reduce bias due to unmeasured confounding. Patients who could not be matched within this caliper range were excluded from further analysis to ensure balanced cohorts suitable for comparative analysis.

After matching, the distribution of the baseline characteristics was compared between the cohorts to evaluate the success of the matching procedure. Special attention was paid to variables known to be associated with secondary causes of NLCs, including comorbid conditions such as endocrine or metabolic disorders, liver insufficiency, chronic alcohol misuse, and the use of cramp-inducing medications (e.g., diuretics, statins, β2-agonists). The goal was to ensure a balanced distribution of known and observable covariates across the treatment groups and thus minimize the selection bias inherent to non-randomized study designs. The quality of matching was assessed by examining the standardized mean differences (SMDs) for each covariate, with values below 0.1 indicating acceptable balance.

The final matched cohorts (QUI and PRI) formed the analytic population for all subsequent outcomes and safety analyses.

### 3.5. Assessment Instruments and Variables

The data for this analysis were extracted from the German Pain e-Registry (GPeR) using a predefined selection of variables directly relevant to the clinical characterization of NLC and the evaluation of treatment response. While the GPeR includes an extensive set of scientifically validated documentation elements and patient-reported outcome measures (PROMs), only a targeted subset of parameters—defined a priori—was used for this study in line with its specific objectives.

All data were originally entered via the iDocLive^®^ platform, a web-based interface enabling time-stamped, structured documentation, plausibility checks, and consistent data quality in routine care settings. The range of self-assessment tools used in specific individual cases and their frequency of use were the responsibility of the treating physicians.

The extracted dataset included sociodemographic and anthropometric information such as age, gender, body weight, body height, and body mass index (BMI, calculated as kg/m^2^). Diagnostic justification was based on ICD-10 codes reflecting the indication for treatment. To describe the natural history and presentation of NLCs, information was collected on the duration of symptoms, the typical temporal pattern (e.g., nocturnal, diurnal, or mixed), the localization of cramps, and the occurrence during daytime.

Symptom burden was further characterized by detailed data on NLC frequency per week, average duration of single NLC episodes (categorized in minutes), and NLC pain intensity, measured on a 0–100 mm visual analog scale (VAS; with 0 = no pain and 100 = worst pain imaginable) and categorized into clinically meaningful severity bands [29].

The functional consequences of NLCs were assessed using the modified pain disability index (mPDI), which evaluates pain-related impairment across key domains of daily life as well as the mPDI subitem #6 regarding the extent of NLC-related sleep problems [30]. Broader aspects of health-related quality-of-life were measured via the Veterans RAND 12-Item Health Survey (VR-12), yielding Physical Component Summary (PCS) and Mental Component Summary (MCS) scores (standardized to a mean of 50 and a standard deviation of 10 in the German population) [31]. General health perception and wellbeing were assessed using the Marburg questionnaire on habitual health findings (MQHHF; NRS35 with 0 = worst restriction possible and 35 = no restriction) [32], while the quality-of-life impairment by pain (QLIP) inventory captured the multidimensional impact of pain beyond functional limitations (NRS40 with 0 = worst impairment possible and 40 = no impairment) [33].

To contextualize symptom severity and guide interpretation of the treatment effects, additional information was collected on the prevalence of comorbid conditions and the presence of pharmacological comedication not specifically directed at NLCs.

All of the extracted parameters formed the basis for the evaluation of baseline comparability, treatment exposure, and clinical response over the predefined 4-week follow-up period. Treatment tolerability and safety were assessed separately and are reported in detail in the corresponding results sections.

### 3.6. Outcome Measures

The primary outcome of the study was the proportion of patients classified as responders based on a predefined composite definition that reflected both clinically meaningful symptom relief and adequate treatment tolerability. To be considered a responder, a patient had to meet five distinct criteria during the final seven days of the 4-week observation period (week 4), relative to the seven days immediately preceding treatment initiation (week − 1). These criteria included a reduction of at least 50% in the cumulative number of nights affected by NLCs, a reduction of at least 50% in the cumulative number of individual NLC episodes, and a reduction of at least 50% in the cumulative duration of NLCs. In addition, responders had to complete the observation period without discontinuing treatment due to lack of efficacy or due to ADRs. Reasons for treatment discontinuation were classified by the treating physician as primarily related to adverse drug reactions or to insufficient therapeutic effect, based on clinical judgment documented at the time of discontinuation.

Each of the five criteria included in the responder definition was also analyzed separately to characterize the individual contribution of each domain to the overall composite response. This approach allowed for a more nuanced understanding of treatment effects, particularly in patients who showed improvement in one or more, but not all, domains.

Secondary outcome measures focused on the absolute and relative changes in the NLC-related parameters from baseline to the end of week 4 and included all of the NLC-related measures mentioned before. Daily symptom tracking of NLC frequency, duration and intensity further allowed for the analysis of within-patient variation and temporal trends throughout the observation period.

Safety and tolerability were evaluated by systematically documenting all ADRs, based on both patient reports and physician assessments. ADRs were categorized according to type, severity, and temporal association with the index therapy. The frequency of ADR-related discontinuations was analyzed as a key marker of treatment tolerability, alongside the rate of discontinuation due to perceived inefficacy. For the purpose of this study, ADRs have been defined as injuries or events related to a drug under investigation (i.e., a relationship to the underlying drug treatment is either possible, probable, or definite). ADRs for the evaluation period were defined as events that were newly reported or reported to worsen in severity after the initiation of any treatment under evaluation and were summarized by treatment groups.

### 3.7. Statistical Analysis

All statistical analyses were conducted using the complete set of anonymized patient data obtained from the GPeR, in accordance with the predefined inclusion and exclusion criteria. The evaluation followed a modified intention-to-treat (mITT) approach, whereby all patients who had received at least one dose of the respective index treatment and had documented at least one post-baseline measurement were included in the analysis. For any outcome involving changes from baseline, only data from patients with both a valid baseline value and a corresponding post-treatment observation were analyzed.

Descriptive and inferential statistics were used to summarize both baseline data and treatment-related changes. For continuous variables, descriptive statistics included the number of patients (*n*), mean, standard deviation (SD), median, minimum, maximum, and 95% confidence interval (CI) for the mean. Categorical and ordinal data were presented as absolute and relative frequencies, including adjusted percentages (a%) where appropriate, with corresponding 95% CIs.

Comparisons between treatment groups were conducted using established statistical procedures. For 2 × 2 contingency tables involving dichotomous variables, McNemar’s test with Edward’s correction was applied. For categorical variables with more than two categories, Pearson’s Chi-squared test was used. Continuous variables were compared using paired samples *t*-tests for normally distributed data, and Wilcoxon’s signed rank test for non-normally distributed data. Where appropriate, effect sizes were estimated using Cohen’s d, and measures of association such as odds ratios (ORs), relative risks (RRs), and numbers needed to treat or harm (NNT/NNH) were calculated.

All tests were conducted using a two-sided significance level of 0.05. Exact *p*-values were reported to a precision of three decimal places; values below 0.001 were denoted as “≤0.001.” As all statistical comparisons were exploratory in nature, no adjustments for multiple testing were applied.

The evaluation of the primary endpoint was based on a sequential non-inferiority and superiority analysis. Non-inferiority of the PRI group (cohort B) relative to the QUI group (cohort A) was confirmed if the lower bound of the 95% confidence interval for the responder rate in the PRI cohort exceeded the corresponding lower bound in the QUI cohort. Following confirmation of non-inferiority, a superiority analysis was subsequently performed if a statistically significant difference (*p* < 0.05) in favor of PRI was observed for the primary endpoint. Superiority was not accepted if the confidence intervals for the primary outcome in both groups overlapped or if the incidence of treatment discontinuations due to ADRs was significantly higher in the PRI cohort.

All descriptive and efficacy analyses were performed using PASW Statistics (version 18.0). The ADRs were coded using MedDRA (version 27.0 2024AA). If multiple diagnoses/symptoms were reported, the corresponding ADRs were split before coding and the ADR frequencies were evaluated both on a patient- and event-based level. Tables and graphs were rendered using Microsoft Excel^®^ for Microsoft 365 MSO (Version 2504 Build 16.0.18730.20122).

### 3.8. Ethical Considerations and Study Registration

This non-interventional, retrospective study was conducted in accordance with the principles of the Declaration of Helsinki and all relevant national and regulatory requirements. Since the study was based on fully anonymized, depersonalized real-world data—with no possibility of tracing participating the patients, physicians, or treatment facilities—approval by an ethics committee was not required.

The study protocol and data evaluation plan were reviewed and approved by the steering committees of both the German Pain Association and the German Pain League. The latter placed specific emphasis on safeguarding patient rights within the framework of this analysis.

All physicians and patients had provided written informed consent upon entry into the GPeR, thereby authorizing the use of their anonymized data for scientific research purposes.

To ensure transparency, the study concept was registered in the European Medicines Agency (EMA) database for non-interventional/epidemiological studies under the identifier EUPAS1000000105. Because this work was a non-interventional/observational retrospective study based on secondary use of existing data, registration in the EMA/ENCePP catalog was undertaken as a transparency measure rather than as a prospective regulatory requirement.

### 3.9. Reporting Standards

The design, conduct, and reporting of this observational study were carried out in accordance with STROBE (Strengthening the Reporting of Observational Studies in Epidemiology) recommendations, ensuring a high level of methodological transparency, reproducibility, and interpretability of the findings [34].

### 3.10. Data Availability

The data supporting the findings of this study were extracted from the GPeR and used under license for the purposes of the present analysis. In compliance with applicable German data protection laws and the European Union General Data Protection Regulation (EU-GDPR), only anonymized and depersonalized patient data were accessed by the research team. All biometric analyses were performed exclusively on these anonymized datasets, as specified in the statistical analysis plan.

Access to individual-level data is restricted due to legal and ethical considerations. The original data are not publicly available and cannot be shared beyond the scope of the current study. Only aggregated and non-identifiable summary data were made available to the study sponsor, and no information permitting the identification of individual participants was ever transmitted or retained beyond the analysis period. All data used for this analysis were permanently deleted after the completion of statistical evaluation.

## 4. Results

### 4.1. Data Selection

**Figure 1:**
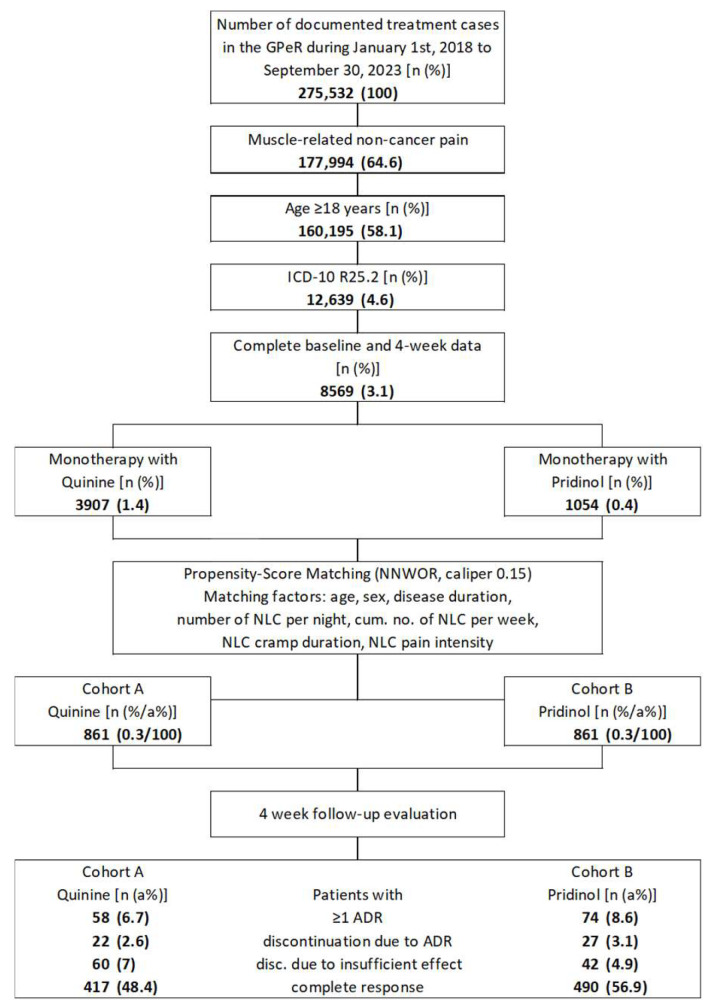
Diagram of patient data selection and flow. Abbreviations: GPeR: German Pain e-Registry; *n*: Number, %: Percent; ICD-10: International Statistical Classification of Diseases and Related Health Problems 10th Revision; R25.2: Cramp and spasm; NNWOR: Nearest neighbor without replacement; NLC: Night leg cramp; a%: Adjusted percent; ADR: Adverse drug reaction.

Based on the in/exclusion criteria and the GPeR population in the evaluation period (1 January 2018 to 30 September 2023; *n* = 275,532), 177,994 datasets (64.6%) were identified in which patients reported on muscle-related non-cancer pain (Figure 1). Of those, 160,195 (58.1%) were 18 years of age or older and 12,639 (4.6%) were coded to suffer from muscle cramp and/or spasm (according to ICD-10 R25.2), and of those 8569 provided evaluable information for at least four weeks using the GPeR documentation standards (enabling an assessment of the safety/tolerability and efficacy of their NLC treatments according to this study concept). Of these, 3907 patients (1.4%) reported monotherapy with QUI, and 1054 (0.4%) with PRI. As a result of the propensity score matching process, the data of 861 patients each (22.0 vs. 81.7a%) were ultimately selected for this analysis and formed the study cohorts A (QUI) and B (PRI).

### 4.2. Patient Demographics and Non-NLC Baseline Characteristics

Comparative analysis of demographic characteristics, as well as NLC-related and non-NLC-related baseline characteristics, confirmed that the two matched cohorts (QUI and PRI) were highly comparable. No statistically significant or biometrically relevant differences were observed between the groups across any of the assessed demographic, clinical, or symptom-related parameters, and the standardized mean difference scores were less than 0.1 for all parameters.

Table 1 summarizes the key demographics and non-NLC-related baseline findings. The mean (±standard deviation) age at treatment initiation for NLCs was 62.6 ± 10.6 years (median 62; range 35–94), with 37.7% of patients being 65 years or older, and 62.3% of patients (*n* = 536) being female. The average BMI was 26.1 ± 4.9 kg/m^2^ and ~one in five patients (20.2 vs. 20.4%) was classified as obese. Comorbidity was common: only 21.6/20.6% of patients in cohorts A/B were free of concurrent medical conditions. On average, patients had 1.9 chronic comorbidities, most frequently involving the musculoskeletal (37.7%), cardiovascular (30.7%), allergic (20.5%), and gastrointestinal (16.1%) systems. More than half of patients were reported to suffer from at least two distinct non-pain comorbidities. Over 88% of patients in both cohorts were on regular non-NLC pharmacological treatments at baseline, with an average of 1.8 ± 1.2 additional drugs prescribed, most commonly targeting cardiovascular, gastrointestinal, genitourinary, or respiratory conditions.

The NLC-related baseline characteristics are shown in Table 2. At a proportion of 76.2% (*n* = 656), most patients reported an NLC history lasting for at least six months, with 62.3% (*n* = 536) experiencing symptoms for at least one year and 44.8% (*n* = 386) experiencing symptoms for more than three years. The majority (58.8%) described their NLCs as sudden-onset pain attacks without intercurrent discomfort, whereas 41.2% reported some degree of ongoing pain in-between.

NLC progression over time was frequently reported: just over half of the patients (50.4% in cohort A and 50.9% in cohort B) stated that the frequency of their NLCs had increased in the past, and a similar proportion (47.6 and 47.3%) reported an increase in pain intensity as well. Additionally, approximately 12.6% of patients noted a qualitative change with respect to their NLC characteristics, while more than half (54.8 and 54.6%) indicated that the overall impact of NLCs on their daily life had worsened over time.

With respect to symptom duration, most patients reported typical NLC episodes lasting between one and five minutes (69.9% in cohort A and 70.4% in cohort B), while around 10% in each cohort reported NLC durations of less than 60 s and approximately 20% reported longer episodes of six minutes or more. The average reported duration of individual NLCs was 4.6 min, with considerable variability (SD 6.7/6.6 min in cohorts A/B; median 2.8 min). NLC frequency was substantial: 26.1% of patients reported multiple NLC episodes per night, and around 40% experienced NLCs at least once nightly. Fewer patients reported NLCs several times per week (approximately 20%) or weekly (around 14%). The average number of NLCs per week was 10.8 ± 10.0, with a median of six episodes.

Although NLCs occurred by definition at night, 18.7% of patients also reported occasional daytime leg cramps. The most frequently affected areas were the calves (84.2%), followed by the thighs (34.5%), feet (23.6%), hamstrings (14.2%), and other areas of lower extremities (7.3%). Nearly half (48.5%) of all patients experienced NLCs in multiple locations.

Pain intensity associated with NLCs was high, with an average peak pain rating of 75.5 ± 16.4 mm on the visual analog scale (VAS), and nearly half of all patients (44.7%) categorized their pain as “strong,” while one quarter rated it as “severe/extreme.”

Functional NLC-related impairment was considerable: patients reported an average of nearly 40 days within the last three months during which they were unable to perform their usual activities, and at 47.6% nearly every other patient in both cohorts reported such limitations on at least 30 days.

Overall disability in daily life as measured with the mPDI confirmed this burden, with mean total scores of 54.8/55.0 mm on the VAS for patients in cohorts A/B, and “moderate” to “strong/extreme” disabilities reported by 60% of patients. NLC-related interference with nighttime sleep as assessed with subitem #6 of the mPDI was even worse (66.9 ± 16.8/17.2 mm VAS) and 42% of patients in both cohorts documented “strong” to “severe/extreme” NLC-related sleep problems.

Quality-of-life measures at baseline indicated significant impairments. The VR-12 physical and mental health summary scores were substantially below population norms (mean PCS/MCS: 37.1/44.0), and average MQHHF scores (mean 15.7) confirmed a clinically relevant decline in perceived wellbeing. Around 64/31% of patients documented VR12 PCS/MCS scores of 40 or less, and 17.2/18.6% of patients in cohort A/B had MQHHF scores ≤ 10, indicating marked overall impairment. NLC-related quality-of-life assessed via the QLIP index was similarly low (mean 18.9), and nearly 70% of patients had QLIP scores in the clinically impaired range.

### 4.3. Treatment Exposure and Adherence

At the end of the 4-week observation period, most patients were still on active treatment with the respective index medication (see Table 3). Specifically, 90.5% of patients in cohort A (QUI) and 92.0% in cohort B (PRI; *n* = 779 and 792, respectively) remained on therapy throughout the full treatment evaluation interval. The most common reason for early treatment discontinuation was a perceived lack of efficacy, which was reported in 7.2% (*n* = 62) of patients receiving QUI and in 5.1% (*n* = 44) of those treated with PRI. Discontinuation due to ADRs was documented in 2.6% (*n* = 22) of patients in cohort A and in 3.1% (*n* = 27) of those in cohort B. Between-group differences in discontinuation rates–whether due to inefficacy or ADRs–were not statistically significant.

The cumulative number of treatment days over the 4-week period was nearly identical between groups, averaging 27.0 ± 3.9 and 27.3 ± 3.3 days in cohorts A and B, with a median of 28 days in both cases.

A notable difference was observed in the cumulative number of tablets taken and the corresponding dosage profiles of the two treatment groups. Patients with QUI reported taking a mean of 31.0 ± 9.7 tablets (median 28; range 1–78), translating into a cumulative dose of 6207.2 ± 1950 mg (median 5600 mg) over four weeks. In contrast, patients with PRI reported a higher mean number of tablets taken, 40.2 ± 19.8 (median 28; range 1–100), which corresponded to a cumulative dose of 120.5 ± 59.3 mg (median 84 mg).

The most common dosage regimen for both medications involved taking one tablet per day (in the evening). Among patients still on treatment at the end of week 4, 83.2% of those in the QUI group and 49.6% of those in the PRI group followed this pattern. The range of daily dosing was narrower for QUI (1–3 tablets/day) compared to PRI (0.5–4 tablets/day). In the QUI group, 80.7% of patients took one tablet daily, 18.1% took two, and only 1.2% took three. In contrast, dosing in the PRI group was more variable: 8.5% of patients took half a tablet, 47.4% took one tablet, 0.8% took one and a half, 33.0% took two, 10.1% took three, and 0.4% took four tablets per day.

With respect to the recommended dosage guidelines for the treatment of NLCs (typically 1–2 tablets before bedtime for both agents), adherence was high in both cohorts. Specifically, 80.7% of patients treated with QUI and 81.1% of those treated with PRI received dosages consistent with label recommendations. Underdosing (i.e., dosages below recommended levels) occurred in none of the QUI patients but was documented in 8.5% of PRI patients, while 19.3% (QUI) and 10.5% (PRI) of patients received doses exceeding recommended values. The cumulative number of daily defined doses (DDDs) taken was 31.0 ± 10/20.1 ± 9.9 for cohort A/B, corresponding to an average number of DDDs of 1.1 ± 0.4/0.7 ± 0.4.

### 4.4. Safety and Tolerability

Over the 28-day treatment period, at least one ADR was reported by 58 patients (6.7%) taking QUI and 74 patients (8.6%) taking PRI (*p* = 0.147; ES: 0.035; see Table 4). In total, 69 ADRs were documented in cohort A and 90 in cohort B. Most of the affected patients experienced only a single ADR (*n* = 51 [5.9%] in cohort A and *n* = 60 [7.0%] in cohort B), while a smaller subset reported two (0.7% vs. 1.4%) or even three ADRs (0.1% vs. 0.2%), respectively. The overall number of ADRs per patient and their distribution did not differ significantly between the treatment groups (*p* = 0.075).

Headache was the most frequently reported ADR in both groups, affecting 2.2/3.5% of patients in cohort A/B, followed by dizziness (0.9% in each cohort), nausea (0.7/1.2%), dry mouth (0.1/1.3%), and abdominal pain (0.1/1.0%). Overall, the ADR profiles differed slightly between cohorts but did not reveal any clinically relevant or systematic deviations from the known safety profiles of the respective agents as reported in the current product characteristics.

Regarding the severity of ADRs, the vast majority were classified as “mild”, accounting for 69.0a% of cases in the QUI cohort and 82.4a% in the PRI cohort. ADRs of “moderate” intensity were reported in 29.3a% and 16.2a% of cases, respectively, and “severe” ADRs occurred in only one patient per group (1.7a% and 1.4a%; *p* = 0.070).

In most instances, no specific ADR-related countermeasures were required. ADRs resolved spontaneously in 53.4a% (QUI) and 47.3a% (PRI) of cases. A smaller number of patients recovered after dose reduction (5.2a% and 12.2%), treatment discontinuation (37.9a% and 36.5a%), or the administration of specific pharmacological interventions (3.4a% and 4.1a%). No hospitalization was necessary in either group. Importantly, the follow-up data confirmed that all of the reported ADRs were resolved completely, with no cases of persistent sequelae or long-term consequences being present.

Despite the similar discontinuation rates, the time to onset of ADRs differed significantly: the mean time to first-reported ADR was 10.2 ± 6.9 days in the QUI cohort (median: day 8; range: 1–27) versus 13.8 ± 6.8 days in the PRI cohort (median: day 13; range: 1–28), indicating a statistically significant difference (*p* = 0.003; ES: 0.533).

### 4.5. Daily Treatment Effects During the 4-Week Evaluation Period

**Figure 2:**
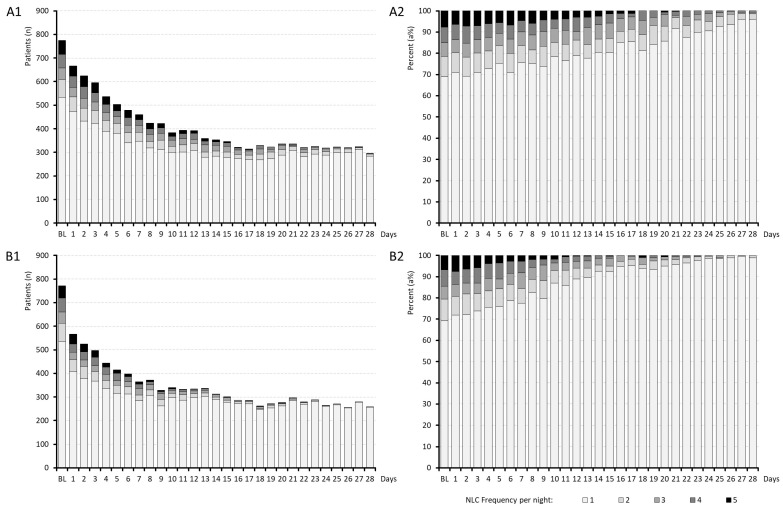
Frequency of night leg cramps. Abbreviations: *n*: Number; NLC: night leg cramps. Note: Panel (**A1**,**A2**): Results for treatment cohort A (quinine); panel (**B1**,**B2**): results for treatment cohort B (pridinol).

**Figure 3:**
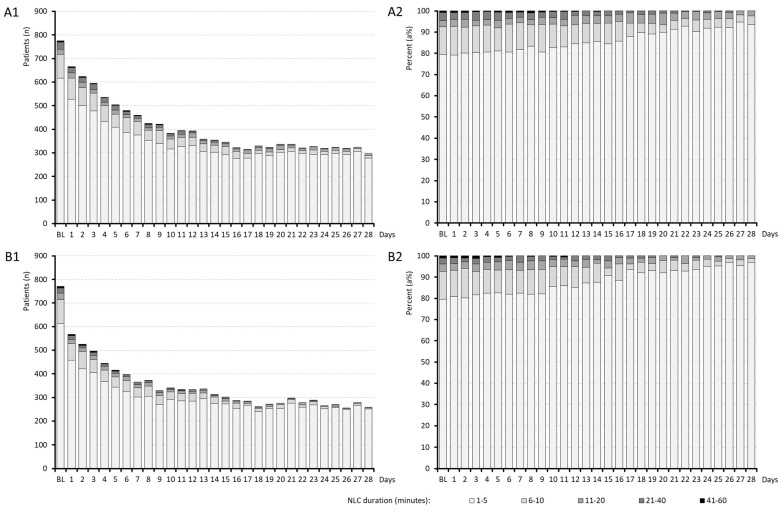
Duration of night leg cramps. Abbreviations: *n*: Number; NLC: night leg cramps. Note: Panel (**A1**,**A2**): Results for treatment cohort A (quinine); panel (**B1**,**B2**): results for treatment cohort B (pridinol).

**Figure 4:**
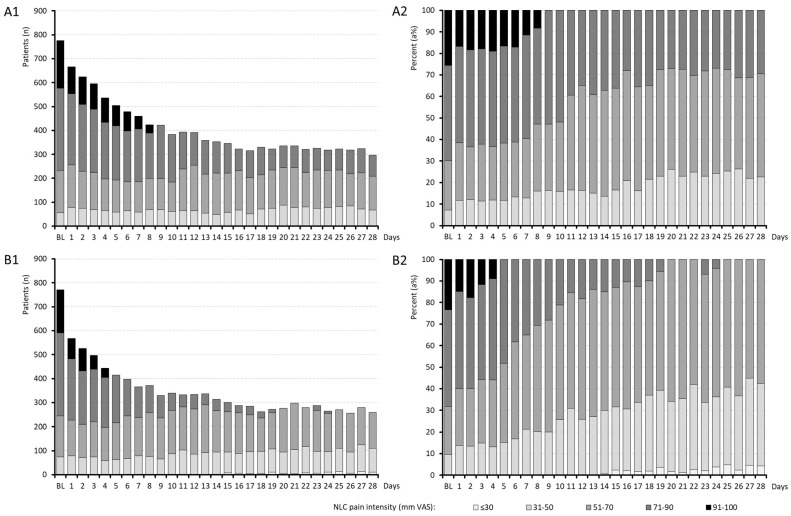
Intensity of night leg cramps. Abbreviations: *n*: Number; NLC: night leg cramps; mm: millimeter; VAS: visual analog scale. Note: Panel (**A1**,**A2**): Results for treatment cohort A (quinine); panel (**B1**,**B2**): results for treatment cohort B (pridinol).

Information on the daily effects of medication on the number of NLCs per night, NLC duration and NLC pain intensity are aggregated in Figure 2, Figure 3 and Figure 4.

At baseline, the occurrence of NLCs was comparable between both treatment cohorts, with 90.0% of patients in the QUI group and 89.5% in the PRI group experiencing at least one NLC-related sleep interruption (see Figure 2). Already during the first night of treatment, the number of NLCs decreased in both cohorts; however, the effect was stronger with PRI vs. QUI since a significantly lower proportion of patients in cohort B vs. A reported NLCs (65.9% vs. 77.4%; *p* < 0.001; OR: 1.8, RR: 1.2). This trend continued consistently over the 4-week treatment period, culminating in NLC occurrence rates of 34.4% in cohort A vs. 30.1% in cohort B on day 28 (*p* = 0.056). In parallel, the cumulative number of nights with NLCs during the whole 4-week evaluation period was significantly lower in cohort B (11.0 ± 2.5) compared to cohort A (12.9 ± 2.6; *p* < 0.001; effect size [ES]: 0.768), indicating a clinically meaningful between-cohort difference. The average event number per night for those who suffered from NLCs decreased from 1.8 ± 1.3 and 1.7 ± 1.3 for cohorts A and B at baseline (*p* = 0.676) to 1.1 ± 0.3 and 1.0 ± 0.1 by the end of week 4 (*p* = 0.023; ES: 0.199). The proportion of patients with more than one NLC per night fell in cohorts A/B from 31.1/30.7% at baseline to 4.1/1.2%, respectively, by day 28, and the cumulative number of NLCs reported by patients over the full 28-day period was 18.6 ± 12.1/14.1 ± 7.3 (*p* < 0.001; ES: 0.445).

The baseline NLC duration was equivalent across cohorts, averaging 4.7 ± 6.9 min (median 2.9; see Figure 3) and thus improving significantly with both treatments (*p* < 0.001). By the end of the evaluation period, the average NLC duration decreased in A/B to 1.8 ± 2.5/1.2 ± 2.0 min (*p* < 0.001 vs. baseline for both; *p* = 0.005 between cohorts; ES: 0.240). By the end of week 4, 63.3% of patients in cohort A versus 47.6% in cohort B reported NLC durations below one minute, and fewer patients with PRI vs. QUI experienced NLCs longer than three minutes (11.6% vs. 20.6%).

The mean NLC pain intensity (shown in Figure 4) at baseline was similar in both groups (cohort A/B: 75.7 ± 16.6/75.6 ± 16.4; *p* = 0.960; ES: 0.003) and decreased until the end of the evaluation period to 61.0 ± 13.0/51.4 ± 11.7 (*p* < 0.001 between groups; ES: 0.780). On day 28, none of the patients in cohort B reported NLC pain intensities > 70 mm on the VAS, whereas this threshold was still exceeded by 29.4% of patients in cohort A (*p* < 0.001).

### 4.6. Primary Endpoint Analyses

**Figure 5:**
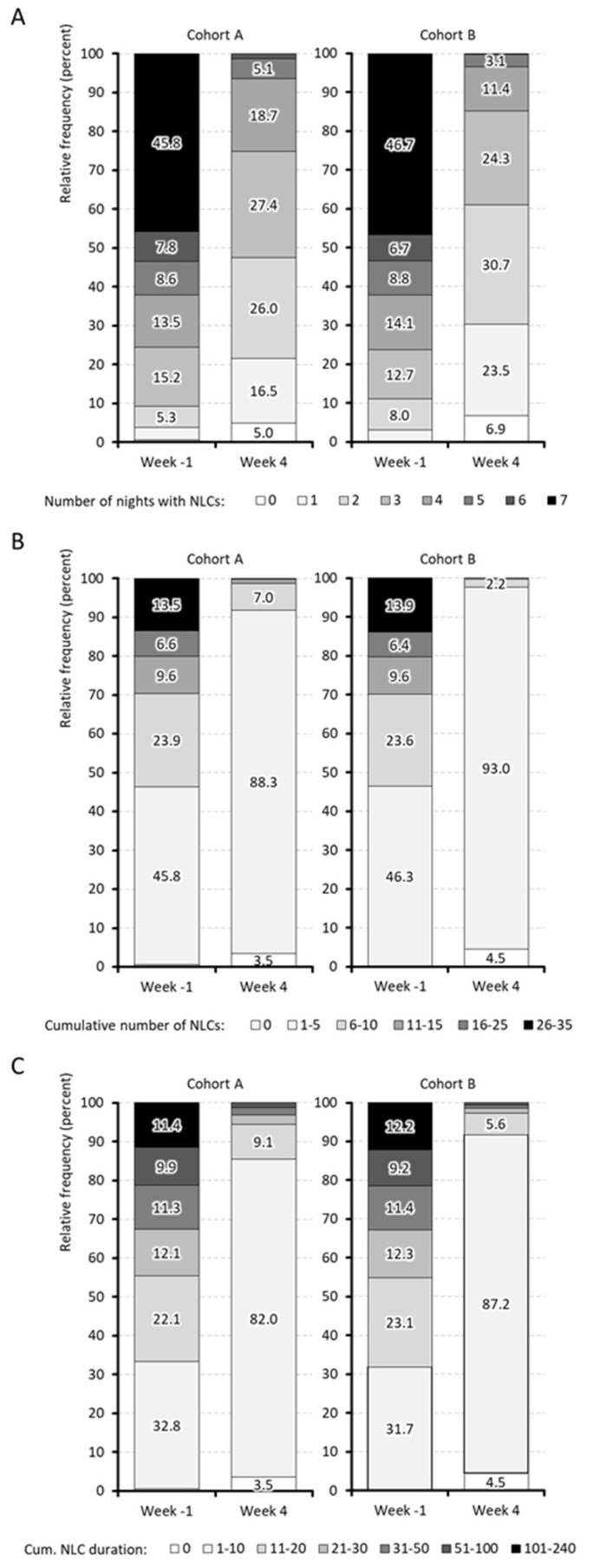
Characteristics of night leg cramps for week − 1 and week 4: (**A**) Number of nights with NLCs, (**B**) Cumulative number of NLCs, (**C**) Cumulative duration of NLCs.

Details on the primary efficacy endpoint components (PECs) are summarized in Table 5 and Table 6 and Figure 5A–C.

### 4.7. PE Component #1: Cumulative Number of Nights with NLC

The cumulative number of nights per week affected by at least one NLC was comparable at baseline (week − 1) in both cohorts, with a total of 5.2 ± 1.9 nights (median 6; range 0–7) in cohort A (QUI) and B (PRI; *p* = 0.911; ES: 0.005). After 4 weeks of treatment, this number significantly decreased to 2.6 ± 1.3 (median 3) in cohort A (*p* < 0.001; ES: 1.604) and 2.2 ± 1.2 (median 2) in cohort B (*p* < 0.001; ES: 1.883). The difference between cohorts for week 4 was statistically significant in favor of PRI (*p* < 0.001; ES: 0.305).

The proportion of patients reporting two or fewer NLC-affected nights per week was comparably low in week − 1 (9.2% vs. 11.0% in cohort A vs. B; *p* = 0.201) but increased significantly at week 4 to 47.5% (cohort A) vs. 61.1% (cohort B), respectively (*p* < 0.001; Figure 5A). The absolute improvement was −2.6 ± 2.3 nights for cohort A and −3.0 ± 2.3 for cohort B (*p* < 0.001; ES: 0.173). The corresponding relative change from baseline was −37.2 ± 58.6% (median − 50%) for cohort A vs. −45.7 ± 53.9% (median − 57%) for cohort B (*p* = 0.002; ES: 0.150). A ≥50% improvement in week 4 vs. week − 1 was reported by 56.2% (A) vs. 65.5% (B; *p* < 0.001) of patients.

### 4.8. PE Component #2: Cumulative Number of NLC Events

The weekly number of cumulative NLC events dropped significantly from 10.2 ± 9.8 to 3.3 ± 2.0 (median 6→3) in cohort A (*p* < 0.001; ES: 0.957) and from 10.2 ± 9.9 to 2.6 ± 1.4 (median 6→2) in cohort B (*p* < 0.001; ES: 1.074), and while baseline figures were comparable, between-group comparison for week 4 showed a significant advantage for PRI vs. QUI (*p* < 0.001; ES: 0.413).

The percentage of patients reporting ≤5 NLCs per week in week 4 was significantly higher in cohort B (97.6%) than in cohort A (91.8%; *p* < 0.001; see Figure 5B). The absolute improvement from baseline was −6.8 ± 9.3 in cohort A vs. −7.6 ± 9.9 in cohort B (*p* = 0.083; ES: 0.084). A reduction of ≥5 NLCs per week was documented by 38.3% of cohort A vs. 42.0% of cohort B (*p* = 0.116), and a ≥50% improvement in week 4 vs. week − 1 was achieved by 57.7% (A) vs. 63.2% (B; *p* = 0.021) of patients.

### 4.9. PE Component #3: Cumulative Duration of NLC

The weekly cumulative NLC duration reduced from 56.8 ± 146.0 in the week before baseline to 6.4 ± 10.3 min (median 16.6→3.3) in week 4 for cohort A (*p* < 0.001; ES: 0.487) and from 53.6 ± 129.5 to 4.3 ± 6.7 min (median 17.3→2.3) for cohort B (*p* < 0.001; ES: 0.538), with significant between-cohort differences for week 4 (*p* < 0.001; ES: 0.242) in favor of PRI vs. QUI.

In the week prior to baseline, the proportion of patients with a ≤10 min cumulative weekly NLC duration was comparable (33.4% vs. 32.2%), but this increased in week 4 to 85.6% in cohort A and 91.9% in cohort B (*p* < 0.001; Figure 5C). The absolute improvement of the cumulative NLC duration was −50.5 ± 138.1 min in cohort A and −49.3 ± 125.0 in cohort B (*p* = 0.860), and a ≥10 min reduction was seen in 54.7% (A) vs. 61.1% (B; *p* = 0.007) of patients. The corresponding relative improvements were −69.4 ± 34.0% (median −79.0%) in cohort A vs. −79.6 ± 22.4% (median − 86.3%) in cohort B (*p* < 0.001; ES: 0.356). A ≥50% improvement in week 4 vs. week − 1 was achieved by 81.3% (A) vs. 91.3% (B; *p* < 0.001) of patients.

### 4.10. PE Components #1–3 Combined: ≥50% Improvement

The number of patients achieving a ≥50% improvement in all three efficacy components (number of NLC-affected nights, frequency, and duration) by week 4 compared to week − 1 differed significantly between cohorts: 49.8% in cohort A (*n* = 429) vs. 58.8% in cohort B (*n* = 506; *p* < 0.001). An improvement in one or two components was reported by 32.5% (A) vs. 32.6% (B) of patients, while 17.7% (A) vs. 8.6% (B) of patients showed no improvement in any of these three components (*p* < 0.001).

### 4.11. PE Components #4 and #5: Treatment Discontinuation Due to ADR or Ineffectiveness

As detailed in the safety section, 2.6% (*n* = 22) of patients in cohort A and 3.1% (*n* = 27) in cohort B discontinued treatment due to an ADR (*p* = 0.865), and 7.2% (*n* = 62) vs. 5.1% (*n* = 44) due to ineffectiveness (*p* = 0.044) (Table 3, Table 4 and Table 6).

### 4.12. Composite Primary Endpoint: Responder Rate

The percentage of patients fulfilling all five criteria of the primary endpoint (≥50% improvement in each of the three core efficacy parameters and no treatment discontinuation due to ADR or ineffectiveness) was significantly higher in cohort B (PRI) compared to cohort A (QUI): 490 patients (56.9%) vs. 417 patients (48.4%; *p* < 0.001). Key biometric indicators for this difference were as follows: odds ratio: 1.41 (95% CI: 1.16–1.70), relative risk: 1.18 (95% CI: 1.07–1.29), number needed to treat (NNT): 12. Therefore, based on the predefined specifications, treatment with PRI under real-world conditions demonstrated not only statistical significance, but also biometrical superiority vs. QUI with respect to the primary composite endpoint.

### 4.13. Secondary Efficacy Analyses

Results of the secondary efficacy analyses performed during this study are shown in Table 7.

**Figure 6:**
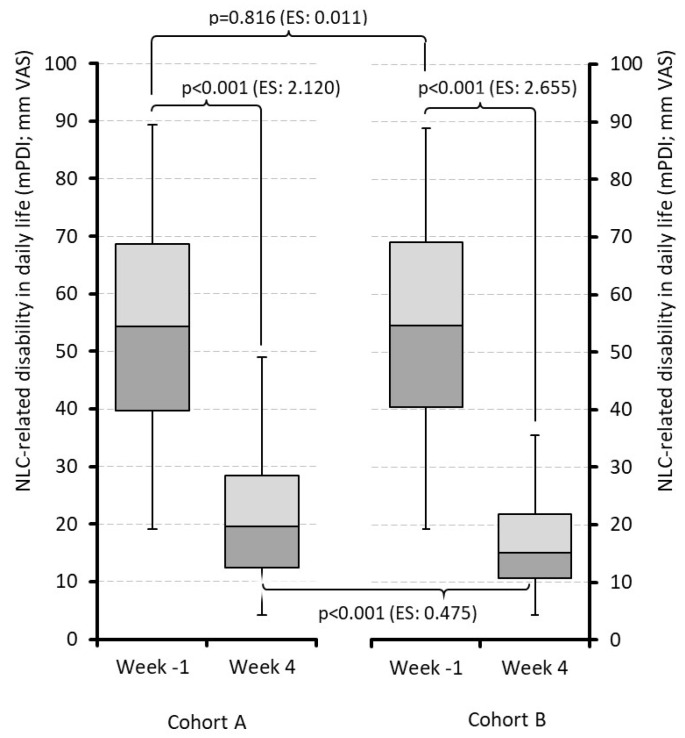
Disability in daily activities due to night leg cramps. Abbreviations: NLC: Night leg cramp; mPDI: modified pain disability index; mm: millimeter; VAS: visual analog scale; ES: effect size. Note: Cohort A: Treatment with quinine; cohort B: treatment with pridinol; week − 1: week before treatment initiation; week 4: last week of treatment evaluation period.

The baseline mPDI scores as a biometrical correlate for NLC-related disabilities in daily life were comparable prior to treatment between the index medications (54.8 ± 19.0 vs. 55.0 ± 18.7; *p* = 0.816) and improved significantly in both cohorts by the end of the 4-week period to 21.4 ± 11.7 (QUI) and 16.6 ± 8.2 (PRI; *p* < 0.001) vs. baseline for both cohorts (*p* < 0.001 between cohorts; ES: 0.475; Figure 6). Absolute improvement reported by patients in cohorts A/B was −33.4 ± 14.1/−38.4 ± 17.6 mm on the VAS (*p* < 0.001; ES: 0.314), and the relative correlates were −61.2 ± 15.0/−67.3 ± 18.4 percent (*p* < 0.001; ES: 0.362). On an individual level, mPDI improvement was achieved in 89.3% (QUI) and 89.4% (PRI) of patients (*p* = 0.938). Only one patient (0.1%) in the PRI group reported a worsening vs. baseline.

**Figure 7:**
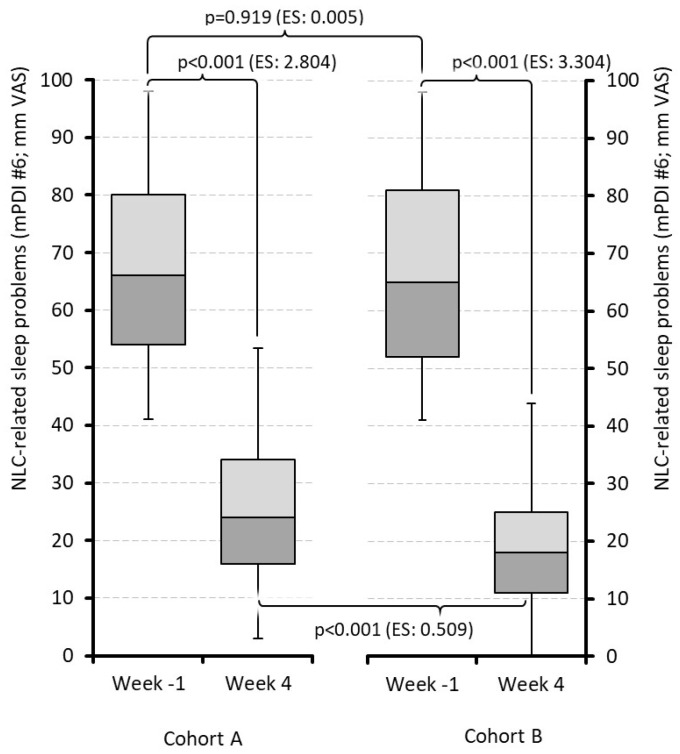
Sleep problems due to night leg cramps. Abbreviations: NLC: Night leg cramp; mPDI#6: subitem number six of the modified pain disability index; mm: millimeter; VAS: visual analog scale; ES: effect size. Note: Cohort A: Treatment with quinine; cohort B: treatment with pridinol; week − 1: week before treatment initiation; week 4: last week of treatment evaluation period.

NLC-related sleep problems reported with subitem #6 of the mPDI—comparable at baseline with 66.9 mm on the VAS on average—improved significantly in both cohorts (A vs. B) until the end of week 4: 25.2 ± 12.6 vs. 19.2 ± 11.0 (*p* < 0.001 for both vs. baseline; *p* < 0.001 for end-of-week-4 data between cohorts, ES: 0.509; Figure 7).

The VR-12 PCS scores were identical at baseline, at 37.1 ± 8.3, and improved significantly until the end of week 4 in both treatment groups, to 43.4 ± 8.5 in cohort A vs. 44.3 ± 8.5 in cohort B (*p* < 0.001 vs. baseline for both; ES: 0.754 vs. 0.851). The absolute improvements were 6.3 ± 1.6 vs. 7.1 ± 1.3 (*p* < 0.001; ES: 0.570), and the relative improvements were 17.9% vs. 20.3% (*p* < 0.001; ES: 0.382). The between-group difference at the end of week 4 was statistically significant in favor of the treatment with PRI (*p* = 0.033; ES: 0.103).

The VR-12 MCS scores improved modestly but significantly in both groups over the 4-week period (i.e., from baseline scores of 44.0 ± 8.3/44.1 ± 8.5 to 46.1 ± 8.3/46.4 ± 8.6; *p* < 0.001 for both). Even though the end-of-week-4 difference between cohorts was not statistically significant (*p* = 0.457), the absolute (2.1 ± 0.8 vs. 2.3 ± 0.8; *p* < 0.001; ES: 0.284) and relative improvements (4.9 ± 2.2% vs. 5.4 ± 2.1%; *p* < 0.001; ES: 0.229) were.

The MQHHF sum scores improved significantly in both cohorts over the treatment period vs. baseline and were 28.2 ± 3.8 for cohort A and 29.8 ± 3.0 for cohort B at the end of week 4 (*p* < 0.001 vs. baseline for both, ES: 2.537 vs. 2.988; *p* < 0.001). The absolute as well as relative improvements vs. baseline were reported to be significantly higher with PRI compared to QUI.

The QLIP scores increased significantly from baseline (18.9 on average for A/B) to the end of week 4 in both groups: 31.6 ± 3.7 (QUI) and 33.3 ± 2.9 (PRI; *p* < 0.001 for both vs. baseline). The end-of-week-4 scores were significantly better with PRI vs. QUI (*p* < 0.001; ES: 0.489); the absolute improvements were 12.8 vs. 14.4 points (*p* < 0.001; ES: 0.415) and the relative gains were 60.6% vs. 68.3% (*p* < 0.001; ES: 0.564).

## 5. Discussion

NLCs are a common and often distressing condition, particularly among older adults. While generally benign and self-limiting in nature, frequent, prolonged and/or severe/intense NLCs can cause significant sleep disruptions and can secondarily impair physical functioning and mental wellbeing.

While non-pharmacological approaches such as hydration, stretching exercises, warm baths, dietary adjustments, or management of underlying conditions may benefit some patients, these measures are often insufficient. Particularly in patients with frequent, persistent NLCs, drug therapy becomes therefore an essential treatment component.

This PRISCILA study investigated the comparative efficacy and safety of two alternative pharmacological treatments—quinine sulfate (QUI) and pridinol mesylate (PRI)—for the management of NLCs. In Germany, both are approved for the treatment of muscle cramps, with differing mechanisms of action. However, due to the lack of randomized controlled trials for PRI, only QUI is listed in national guidelines for the treatment of muscle cramps [14]. Given the multifactorial and heterogeneous nature of NLC pathophysiology, a uniformly effective treatment is unlikely, further underscoring the need for comparative effectiveness data to guide clinical decisions. However, the absence of randomized controlled trials for PRI limits the level of evidence currently available for direct comparative conclusions.

The present study addressed this gap by evaluating two treatments in two large, comparably affected cohorts over a 4-week period. The findings demonstrate that both QUI and PRI were able to alleviate NLCs in affected patients as they significantly reduced all core NLC-related parameters: the frequency of nights with NLCs, the number of NLC episodes, and the NLC duration and intensity. These improvements translated into tangible clinical benefits, including reductions in NLC-related disability, better sleep-related physical and mental quality-of-life, and improved overall wellbeing. Importantly, both therapies were generally well tolerated. Adverse drug reactions were infrequent, mild in most cases, and rarely led to discontinuation. Only 6.7% of patients treated with QUI and 8.6% of those on PRI reported ADRs, and premature treatment cessation due to ADRs occurred in only 2.6% and 3.1% of patients, respectively. This safety profile, in combination with the observed clinical responses, supports the use of both medications for the short-term therapeutic management of patients with significant NLC symptomatology under routine care conditions.

However, direct comparison between the two agents revealed measurable differences in short-term treatment outcomes. While both treatments led to clinically meaningful improvements across core NLC-related parameters, PRI was associated with numerically larger reductions in NLC frequency, intensity, and duration, as well as greater improvements in NLC-related disability and quality-of-life measures. A higher proportion of patients in the PRI group fulfilled all criteria of the predefined composite primary endpoint, including clinically relevant improvement across efficacy domains without treatment discontinuation. Although statistically significant, the absolute difference in responder rates was moderate, corresponding to a number needed to treat of 12. From a clinical perspective, these findings suggest an incremental short-term advantage of PRI over QUI under real-world conditions rather than a transformative treatment effect.

Both pharmacotherapies were associated with clinically relevant improvements and acceptable short-term tolerability in this real-world setting. However, the available data are not sufficient to support a preferential first-line positioning of PRI over QUI. While QUI has demonstrated efficacy and safety in several randomized, placebo-controlled trials, evidence for PRI is currently limited to observational data. Accordingly, PRI may represent a reasonable therapeutic option in patients who do not respond adequately to QUI, do not tolerate it, or in whom its use is contraindicated, rather than a replacement for established first-line approaches.

Thus, the PRISCILA study contributes not only meaningful evidence to the current understanding of pharmacological NLC management, highlighting the need for individualized treatment approaches in a condition that is often underestimated in terms of its impact on patients’ wellbeing, but also—as a side effect—the results of this analysis emphasize the significant clinical burden of this condition. Despite often being regarded as minor or transient, the frequency, intensity, and duration of NLCs in the studied population clearly demonstrate their potential to severely affect quality-of-life. In addition, patients reported significant sleep disturbance and daytime fatigue, highlighting the need for broader effective therapeutic strategies such as QUI and PRI. These observations further emphasize the need for adequately powered controlled studies to better define optimal treatment strategies for patients with clinically relevant NLC.

### 5.1. Strengths and Limitations

This study offers important insights into the comparative effectiveness and tolerability of QUI and PRI in the real-world management of NLCs. Several strengths and limitations should, however, be considered when interpreting the findings.

### 5.2. Strengths

A key strength of this study lies in its real-world design, which allows for the evaluation of treatment effects under routine care conditions, reflecting the realities of clinical decision-making in everyday practice. This enhances the external validity and generalizability of the findings, especially in the absence of high-quality head-to-head comparisons between the two agents. Due to the completely non-interventional nature of the retrospective analysis of depersonalized data from standard care, the usual (negative) phenomena of uncontrolled studies (such as the Hawthorne effect, etc.) were avoided and the evaluation of patient-relevant effects was ensured.

Second, the study includes a large sample size, enabling robust statistical analyses. The patient populations in both treatment groups were carefully selected based on clearly defined inclusion and exclusion criteria and subsequently harmonized using propensity score matching. This methodological approach helped to minimize the impact of potential baseline differences and confounding factors, thereby increasing the credibility of the results, especially with respect to the between-group comparisons reported.

Third, the study comprehensively assessed a wide spectrum of clinically relevant outcomes, including both objective NLC-related parameters (e.g., number, duration and intensity of NLC) and patient-reported measures (e.g., physical and mental quality-of-life, NLC-related quality-of-life impairments; disability, and wellbeing). This multidimensional evaluation offers a more holistic picture of treatment effects, aligning with patient-centered care principles.

Lastly, the analysis followed a pre-specified statistical plan, applied appropriate significance testing, and reported effect sizes alongside *p*-values, supporting transparency and reproducibility.

### 5.3. Limitations

The most important limitation of this study is its retrospective, non-interventional, and non-randomized design. As is inherent in real-world data collection, there is no control over the treatment allocation, which may be subject to indication bias (e.g., the decision to prescribe PRI versus QUI may have been influenced by clinical judgment or prior treatment experiences, which in turn could have affected outcomes, etc.).

Although propensity score matching was applied to account for relevant baseline characteristics and minimize selection bias, residual confounding cannot be entirely excluded. In particular, certain potentially relevant clinical variables such as laboratory parameters (e.g., electrolyte status), formally diagnosed sleep disorders, or the use of non-prescription supplements (e.g., magnesium) were not systematically available in the registry and could therefore not be included in the matching model. Additionally, since the study was not blinded, patients were aware of the treatment they were receiving, and expectation effects may have contributed to some of the patient-reported outcomes.

Another limitation relates to the use of patient-reported outcome measures (PROMs) via self-reporting instruments, which—although scientifically validated and highly relevant from a clinical perspective—carry a certain risk of subjective misinterpretation, reporting errors, or recall bias. Furthermore, the data were obtained in an uncontrolled setting, and treatment adherence was based on self-reporting, which may limit the precision of the exposure assessment.

Finally, this study was not designed to replace randomized controlled trials but rather to complement them by providing insights from real-life settings. While the results indicate a modest short-term advantage of PRI over QUI in routine care, they should be interpreted within the limitations of the study design and considered hypothesis-generating rather than conclusive. Moreover, the predefined 4-week observation period does not allow assessment of rare, delayed, or cumulative adverse events, particularly those historically associated with quinine; absence of serious adverse drug reactions in this analysis should therefore not be interpreted as evidence of long-term safety equivalence.

## 6. Conclusions

In this large, real-world comparative effectiveness study, both QUI and PRI were associated with clinically meaningful improvements in the management of NLCs. Across a broad range of patient-reported and objective outcome measures—including NLC frequency, duration, intensity, and impact on quality-of-life—both treatments showed consistent short-term effectiveness and acceptable tolerability over a 4-week period of routine use. Within this analysis, PRI performed at least comparably to QUI across most of the evaluated dimensions and was associated with modestly more favorable short-term outcomes in selected parameters, including achievement of the composite primary endpoint and improvements in physical disability, pain-related quality-of-life, and subjective wellbeing. These findings suggest that PRI may represent a reasonable therapeutic option for patients with moderate-to-severe NLCs, particularly in those who do not respond adequately to, do not tolerate, or cannot receive established pharmacological treatments.

While the retrospective design and inherent limitations of real-world data require cautious interpretation, the consistency of the observed effects across the well-matched cohorts supports the clinical relevance of these findings in routine care settings. Rather than providing definitive treatment recommendations, the results may inform individualized therapeutic decision-making and contribute to discussions on pharmacological sequencing in patients with persistent or burdensome NLCs.

Future prospective, randomized studies are warranted to confirm these observations and to assess the long-term safety, adherence, and predictors of response for both medications. Until such data are available, the present analysis contributes additional real-world evidence to the symptomatic treatment of NLCs and suggests that more than one pharmacological option may be available for patients in whom preventive or non-pharmacological measures are insufficient.

## Figures and Tables

**Table 1:** Demographic and baseline characteristics not related to night leg cramps.

	Cohort A	(QUI)	Cohort B	(PRI)	Between-Cohort Differences	
Number of patients [*n* (%)]	861	(100)	861	(100)	Significance	(Effect size)
Age * [years; mean (SD)]		62.6	(10.6)		1.000	(0.000)
Range [years; min–max (median)]		35–94 (62)				
Age ≥ 65 years [*n* (%)]		325	(37.7)			
≥75 years [*n* (%)]		114	(13.2)			
≥85 years [*n* (%)]		24	(2.8)			
Female gender * [*n* (%)]		536	(62.3)		1.000	(0.000)
Body mass index [BMI; kg/m^2^; mean (SD)]	26.1	(4.9)	26.1	(4.9)	0.962	(0.002)
Range [min–max]	14.0–43.7		13.9–43.1			
Obesity grade 1–3 [*n* (%)]	174	(20.2)	176	(20.4)		
Comorbidities [*n*; mean (SD)]	1.9	(1.7)	1.9	(1.7)	0.609	(0.025)
Number of concurrent comorbidities: None [*n* (%)]	186	(21.6)	177	(20.6)		
One [*n* (%)]	239	(27.8)	237	(27.5)		
Two [*n* (%)]	180	(20.9)	181	(21)		
Three [*n* (%)]	111	(12.9)	116	(13.5)		
≥Four [*n* (%)]	145	(16.8)	150	(17.4)		
Comorbidities: Musculoskeletal system [*n* (%)]	325	(37.7)	324	(37.6)		
Cardiac system [*n* (%)]	263	(30.5)	265	(30.8)		
Allergy [*n* (%)]	175	(20.3)	177	(20.6)		
Gastrointestinal tract [*n* (%)]	138	(16)	139	(16.1)		
Skin [*n* (%)]	107	(12.4)	109	(12.7)		
Psychological disorders [*n* (%)]	106	(12.3)	106	(12.3)		
Pulmonary system [*n* (%)]	103	(12)	106	(12.3)		
Metabolism, endocrine system [*n* (%)]	100	(11.6)	102	(11.8)		
Urinary tract [*n* (%)]	78	(9.1)	79	(9.2)		
Liver [*n* (%)]	60	(7)	64	(7.4)		
Blood [*n* (%)]	52	(6)	53	(6.2)		
Nervous system [*n* (%)]	42	(4.9)	46	(5.3)		
Other [*n* (%)]	64	(7.4)	69	(8)		
Concurrent pharmacological non-NLC treatments [*n*; mean (SD)]	1.8	(1.2)	1.8	(1.3)	0.892	(0.007)
Number of concurrent pharmacological non-NLC treatments: None [*n* (%)]	102	(11.8)	100	(11.6)		
One [*n* (%)]	283	(32.9)	281	(32.6)		
Two [*n* (%)]	246	(28.6)	263	(30.5)		
Three [*n* (%)]	148	(17.2)	125	(14.5)		
≥Four [*n* (%)]	82	(9.5)	92	(10.7)		
Non-NLC comedication: Cardiovascular system [*n* (%)]	349	(40.5)	352	(40.9)		
Alimentary tract and metabolism [*n* (%)]	203	(23.6)	189	(22)		
Genitourinary tract/sex hormones [*n* (%)]	176	(20.4)	203	(23.6)		
Respiratory system [*n* (%)]	180	(20.9)	177	(20.6)		
Systemic hormonal preparations [*n* (%)]	135	(15.7)	132	(15.3)		
Musculoskeletal system [*n* (%)]	136	(15.8)	117	(13.6)		
Nervous system [*n* (%)]	123	(14.3)	123	(14.3)		
Dermatologicals [*n* (%)]	97	(11.3)	106	(12.3)		
Blood and blood-forming organs [*n* (%)]	66	(7.7)	68	(7.9)		
Other [*n* (%)]	114	(13.2)	119	(13.8)		

Abbreviations: NLC: Night leg cramps; *n*: number; %: percent; QUI: quinine; PRI: pridinol; SD: standard deviation; kg: kilogram, m^2^: square meter. Note: Parameters marked with an asterisk (*) are those used for the propensity score-based cohort matching.

**Table 2:** NLC baseline characteristics.

	Cohort A	(QUI)	Cohort B	(PRI)	Between-Cohort Differences	
Number of patients [*n* (%)]	861	(100)	861	(100)	Significance	(Effect size)
NLC disease duration *: ≤6 months [*n* (%)]		205	(23.8)		1.000	(0.000)
7–12 months [*n* (%)]		120	(13.9)			
2–3 years [*n* (%)]		150	(17.4)			
>3 years [*n* (%)]		386	(44.8)			
NLC pattern *: pain attacks, pain-free in-between [*n* (%)]		506	(58.8)		1.000	(0.000)
Pain attacks, with pain in-between [*n* (%)]		355	(41.2)		1.000	(0.000)
NLC attack duration * [minutes; mean (SD)]	4.6	(6.7)	4.6	(6.6)	0.865	(0.008)
NLC duration: <1 min. [*n* (%)]	86	(10)	84	(9.8)		
1–2 min. [*n* (%)]	251	(29.2)	248	(28.8)		
3–5 min. [*n* (%)]	351	(40.8)	358	(41.6)		
6–10 min. [*n* (%)]	112	(13)	112	(13)		
>10 min. [*n* (%)]	61	(7.1)	59	(6.9)		
NLC frequency * [number per week; mean (SD)]	10.8	(10.0)	10.9	(10.0)	0.891	(0.007)
Multiple NLCs per night [*n* (%)]	225	(26.1)	225	(26.1)		
One NLC per night [*n* (%)]	340	(39.5)	345	(40.1)		
Several NLCs per week [*n* (%)]	172	(20)	170	(19.7)		
One or less per week [*n* (%)]	124	(14.4)	121	(14.1)		
Daytime cramps [*n* (%)]	161	(18.7)	161	(18.7)	1.000	(0.000)
NLC localization *: Calves [*n* (%)]		725	(84.2)		1.000	(0.000)
Thighs [*n* (%)]		297	(34.5)			
Feet [*n* (%)]		203	(23.6)			
Hamstrings [*n* (%)]		122	(14.2)			
Other [*n* (%)]		63	(7.3)			
Concurrent affected localizations [*n*; mean (SD)]		1.6	(0.8)		1.000	(0.000)
One [*n* (%)]		443	(51.5)			
Two [*n* (%)]		312	(36.2)			
Three or more [*n* (%)]		106	(12.3)			
NLC pain intensity * [mm VAS; mean (SD)]		75.5	(16.4)		0.996	(0.000)
NLC intensity *: Mild [31–50 mm VAS; *n* (%)]	60	(7)	60	(7)		
Moderate [51–70 mm VAS; *n* (%)]	203	(23.6)	204	(23.7)		
Strong [71–90 mm VAS; *n* (%)]	384	(44.6)	386	(44.8)		
Severe/extreme [91–100 mm VAS; *n* (%)]	214	(24.9)	211	(24.5)		
Days usual activities could not be performed due to NLCs in the last three months [cum. *n*; mean (SD)]	39.5	(32.2)	39.3	(31.9)	0.905	(0.006)
0–10 [*n* (%)]	234	(27.2)	236	(27.4)		
11–20 [*n* (%)]	113	(13.1)	107	(12.4)		
21–30 [*n* (%)]	104	(12.1)	102	(11.8)		
31–50 [*n* (%)]	129	(15)	122	(14.2)		
51–70 [*n* (%)]	88	(10.2)	112	(13)		
>70 [*n* (%)]	193	(22.4)	182	(21.1)		
NLC-related disability in daily life activities [mPDI; mm VAS; mean (SD)]	54.8	(19.0)	55.0	(18.7)	0.816	(0.011)
None [≤30 mm VAS; *n* (%)]	91	(10.6)	85	(9.9)		
Mild [31–50 mm VAS; *n* (%)]	252	(29.3)	252	(29.3)		
Moderate [51–70 mm VAS; *n* (%)]	321	(37.3)	327	(38)		
Strong [71–90 mm VAS; *n* (%)]	178	(20.7)	180	(20.9)		
Severe/extreme [91–100 mm VAS; *n* (%)]	19	(2.2)	17	(2)		
NLC-related disability sleep problems [mPDI#6; mm VAS; mean (SD)]	66.9	(16.8)	66.9	(17.2)	0.919	(0.005)
None [≤30 mm VAS; *n* (%)]	4	(0.5)	4	(0.5)		
Mild [31–50 mm VAS; *n* (%)]	162	(18.8)	167	(19.4)		
Moderate [51–70 mm VAS; *n* (%)]	332	(38.6)	324	(37.6)		
Strong [71–90 mm VAS; *n* (%)]	264	(30.7)	251	(29.2)		
Severe/extreme [91–100 mm VAS; *n* (%)]	99	(11.5)	115	(13.4)		
Quality-of-life—physical dimension [VR12-PCS; mean (SD)]	37.1	(8.3)	37.1	(8.3)	0.949	(0.003)
VR12-PCS: ≤30 [*n* (%)]	180	(20.9)	182	(21.1)		
31–40 [*n* (%)]	374	(43.4)	372	(43.2)		
41–50 [*n* (%)]	262	(30.4)	262	(30.4)		
>50 [*n* (%)]	45	(5.2)	45	(5.2)		
Quality-of-life—mental dimension [VR12-MCS; mean (SD)]	44.0	(8.3)	44.1	(8.5)	0.853	(0.009)
VR12-PCS: ≤30 [*n* (%)]	40	(4.6)	47	(5.5)		
31–40 [*n* (%)]	226	(26.2)	218	(25.3)		
41–50 [*n* (%)]	383	(44.5)	378	(43.9)		
>50 [*n* (%)]	212	(24.6)	218	(25.3)		
Overall wellbeing [MQHHF, NRS35; mean (SD)]	15.7	(5.8)	15.7	(5.9)	0.867	(0.008)
Overall wellbeing significantly impaired [MQHHF ≤ 10; *n* (%)]	148	(17.2)	160	(18.6)	0.451	(0.018)
Quality-of-life impairment by pain [QLIP; mean (SD)]	18.9	(4.2)	18.9	(3.9)	0.881	(0.007)
Clinically relevant impairment [QLIP < 20; *n* (%)]	591	(68.6)	598	(69.5)	0.715	(0.009)

Abbreviations: NLC: Night leg cramp; *n*: number; %: percent; QUI: quinine; PRI: pridinol; SD: standard deviation; min.: minutes; mm: millimeter; VAS: visual analog scale; mPDI: modified pain disability index; mPDI#6: subitem number 6 of the mPDI; VR12: Veterans Rand quality-of-life questionnaire, short form 12; PCS/MCS: physical and mental component scores of the VR12; MQHHF: Marburg questionnaire on habitual health findings; QLIP: quality-of-life impairment by pain inventory. Note: Parameters marked with an asterisk (*) are those used for the propensity score-based cohort matching.

**Table 3:** Treatment data.

	Cohort A	(QUI)	Cohort B	(PRI)	Between-Cohort Differences	
Number of patients [*n* (%)]	861	(100)	861	(100)	Significance	(Effect size)
Patients on treatment at end of week 4 [*n* (%)]	779	(90.5)	792	(92)	0.268	(0.027)
Discontinuation due to ADR [*n* (%)]	22	(2.6)	27	(3.1)	0.469	(0.017)
Inadequate (low) efficacy [*n* (%)]	62	(7.2)	44	(5.1)	0.066	(0.044)
Cumulative number of days with treatment [cum. *n*; mean (SD)]	27.0	(3.9)	27.3	(3.3)	0.159	(0.075)
Cumulative dose taken [mg; mean (SD)]	6207.0	(1950)	120.5	(59.3)	na	na
Cumulative number of tablets taken [*n*; mean (SD)]	31.0	(9.7)	40.2	(19.8)	<0.001	(0.586)
Cumulative number of DDDs taken [*n*; mean (SD)]	31.0	(10)	20.1	(9.9)	<0.001	(1.116)
Average number of DDDs taken per day [*n*; mean (SD)]	1.1	(0.4)	0.7	(0.4)		

Abbreviations: *n*: Number; %: percent; QUI: quinine; PRI: pridinol; ADR: adverse drug reaction; SD: standard deviation; mg: milligram; DDDs: defined daily doses; na: not applicable.

**Table 4:** Safety and tolerability data.

	Cohort A	(QUI)	Cohort B	(PRI)	Between-Cohort Differences	
Number of patients [*n* (%)]	861	(100)	861	(100)	Significance	(Effect size)
Number of ADRs reported [*n*]	69		90			
Patients with ≥1 ADR [*n* (%)]	58	(6.7)	74	(8.6)	0.147	(0.035)
Patients with ≥2 ADRs [*n* (%)]	7	(0.8)	14	(1.6)	0.124	(0.037)
ADR occurrence [treatment day; mean (SD)]	10.2	(6.9)	13.8	(6.8)	0.003	(0.533)
ADR occurrence in treatment week 1 [*n* (%)]	27	(3.1)	13	(1.5)		
2 [*n* (%)]	18	(2.1)	31	(3.6)		
3 [*n* (%)]	8	(0.9)	18	(2.1)		
4 [*n* (%)]	5	(0.6)	12	(1.4)		
Preferred MedDRA ADR terms: Headaches [*n* (%)]	19	(2.2)	30	(3.5)	0.111	(0.038)
Dizziness [*n* (%)]	8	(0.9)	8	(0.9)	1.000	(0.000)
Nausea [*n* (%)]	6	(0.7)	10	(1.2)	0.315	(0.024)
Dry mouth [*n* (%)]	1	(0.1)	11	(1.3)	0.004	(0.070)
Abdominal pain [*n* (%)]	1	(0.1)	9	(1)	0.011	(0.061)
Fatigue [*n* (%)]	4	(0.5)	6	(0.7)	0.526	(0.015)
Weakness/asthenia [*n* (%)]	4	(0.5)	6	(0.7)	0.526	(0.015)
Circulatory reaction [*n* (%)]	1	(0.1)	6	(0.7)	0.058	(0.046)
Hypotension [*n* (%)]	3	(0.3)	3	(0.3)	1.000	(0.000)
Itching [*n* (%)]	6	(0.7)	0	(0)	0.014	(0.059)
Sweating [*n* (%)]	6	(0.7)	0	(0)	0.014	(0.059)
Skin rash [*n* (%)]	5	(0.6)	0	(0)	0.025	(0.054)
Vomiting [*n* (%)]	3	(0.3)	0	(0)	0.083	(0.042)
Tachycardia [*n* (%)]	1	(0.1)	1	(0.1)	1.000	(0.000)
Loss of appetite [*n* (%)]	1	(0.1)	0	(0)	0.317	(0.024)
MedDRA SOC: Nervous system disorders [*n* (%)]	27	(3.1)	38	(4.4)	0.164	(0.034)
Gastrointestinal disorders [*n* (%)]	11	(1.3)	30	(3.5)	0.003	(0.072)
General disorders and administration site conditions [*n* (%)]	8	(0.9)	12	(1.4)	0.368	(0.022)
Vascular disorders [*n* (%)]	4	(0.5)	9	(1)	0.164	(0.034)
Skin and subcutaneous tissue disorders [*n* (%)]	17	(2)	0	(0)	<0.001	(0.100)
Cardiac disorders [*n* (%)]	1	(0.1)	1	(0.1)	1.000	(0.000)
Metabolism and nutrition disorders [*n* (%)]	1	(0.1)	0	(0)	0.317	(0.024)
Countermeasures: None [*n* patients (%)]	31	(3.6)	35	(4.1)	0.616	(0.012)
Dose reduction [*n* patients (%)]	3	(0.3)	9	(1)	0.082	(0.042)
Treatment discontinuation [*n* patients (%)]	22	(2.6)	27	(3.1)	0.865	(0.017)
Drug treatment [*n* patients (%)]	2	(0.2)	3	(0.3)	0.654	(0.011)

Abbreviations: *n*: Number; %: percent; QUI: quinine; PRI: pridinol; ADR: adverse drug reaction; SD: standard deviation; MedDRA: medical dictionary for regulatory activities; SOC: system of organ classes. Notes: Shown are the data for the whole 4-week evaluation period.

**Table 5:** Comparison of week − 1 vs. week 4 characteristics of night leg cramps.

	Cohort A	(QUI)	Cohort B	(PRI)	Between-Cohort Differences	
Number of patients [*n* (%)]	861	(100)	861	(100)	Significance	(Effect size)
Cumulative number of nights with NLCs per week in week − 1 [*n*; mean (SD)]	5.2	(1.9)	5.2	(1.9)	0.911	(0.005)
Week 4 [*n*; mean (SD)]	2.6	(1.3)	2.2	(1.2)	<0.001	(0.305)
Difference absolute [*n*; mean (SD)]	−2.6	(2.3)	−3.0	(2.3)	<0.001	(0.173)
Relative [percent; mean (SD)]	−37.2	(58.6)	−45.7	(53.9)	0.002	(0.150)
Within-cohort difference (significance (effect size))	<0.001	(1.604)	<0.001	(1.883)		
Cumulative number of NLCs per week in week −1 [*n*; mean (SD)]	10.2	(9.8)	10.2	(9.9)	0.870	(0.008)
Week 4 [*n*; mean (SD)]	3.3	(2.0)	2.6	(1.4)	<0.001	(0.413)
Difference absolute [*n*; mean (SD)]	−6.8	(9.3)	−7.6	(9.9)	0.083	(0.084)
Relative [percent; mean (SD)]	−35.1	(68.0)	−44.3	(64.1)	0.004	(0.140)
Within-cohort difference (significance (effect size))	<0.001	(0.957)	<0.001	(1.074)		
Cumulative NLC duration in week − 1 [minutes; mean (SD)]	56.8	(146.0)	53.6	(129.5)	0.631	(0.023)
Week 4 [minutes; mean (SD)]	6.4	(10.3)	4.3	(6.7)	<0.001	(0.242)
Difference absolute [minutes; mean (SD)]	−50.5	(138.1)	−49.3	(125.0)	0.860	(0.009)
Relative [percent; mean (SD)]	−69.4	(34.0)	−79.6	(22.4)	<0.001	(0.356)
Within-cohort difference (significance (effect size))	<0.001	(0.487)	<0.001	(0.538)		

Abbreviations: *n*: Number; %: percent; QUI: quinine; PRI: pridinol; NLC: night leg cramp; SD: standard deviation.

**Table 6:** Primary endpoint.

Primary Endpoint Component	PEC #1	PEC #2	PEC #3	PEC #4	PEC #5	Primary Endpoint
Description	50% reduction of cum. nights with NLC	50% reduction of cum. number of NLC	50% reduction of cum. duration of NLC	No ADR-related treatment discontinuation	No discontinuation due to low efficacy	All PEC (#1–#5) fulfilled
Cohort A [QUI; *n* (%; 95% CI)]	484 (56.2 52.9–59.5)	497 (57.7; 54.4–61.0)	700 (81.3; 78.5–83.8)	839 (97.4; 96.2–98.3)	799 (92.8; 90.9–94.3)	417 (48.4; 45.1–51.8)
Cohort B [PRI; *n* (%; 95% CI)]	564 (65.5; 62.3–68.6)	544 (63.2; 59.5–66.3)	786 (91.3; 89.2–93.0)	834 (96.9; 95.5–97.8)	817 (94.9; 93.2–96.2)	490 (56.9; 53.6–60.2)
Significance	*p* < 0.001	*p* = 0.021	*p* < 0.001	*p* = 0.865	*p* = 0.071	*p* < 0.001
Odds ratio (95% CI)	1.48 (1.22–1.80)	1.26 (1.04–1.53)	2.41 (1.80–3.23)	0.81 (0.46–1.43)	1.44 (0.97–2.15)	1.41 (1.16–1.70)
Relative risk (95% CI)	1.17 (1.08–1.26)	1.10 (1.01–1.18)	1.12 (1.08–1.17)	0.81 (0.47–1.42)	1.02 (1.00–1.05)	1.18 (1.07–1.29)
NNT (PEC #1–3)/NNH (PEC #4–5)	11	18	10	172	48	12

Abbreviations: PEC: Primary endpoint component; NLC: night leg cramp/s; ADR: adverse drug reaction; *n*: number; %: percent; 95% CI: 95 percent confidence interval; NNT: number needed to treat; NNH: number needed to harm.

**Table 7:** Treatment effects.

	Cohort A	(QUI)	Cohort B	(PRI)	Between-Cohort Differences	
Number of patients [*n* (%)]	861	(100)	861	(100)	Significance	(Effect size)
Disability in daily life activities (mPDI) at baseline [mm VAS; mean (SD)]	54.8	(19.0)	55.0	(18.7)	0.816	(0.011)
At end of week 4 [mm VAS; mean (SD)]	21.4	(11.7)	16.6	(8.2)	<0.001	(0.475)
Difference absolute [mm VAS; mean (SD)]	−33.4	(14.1)	−38.4	(17.6)	<0.001	(0.314)
Relative [percent; mean (SD)]	−61.2	(15.0)	−67.3	(18.4)	<0.001	(0.362)
Within-cohort difference (significance (effect size))	<0.001	(2.120)	<0.001	(2.655)		
Sleep problems (mPDI#6) at baseline [mm VAS; mean (SD)]	66.9	(16.8)	66.9	(17.2)	0.919	(0.005)
At end of week 4 [mm VAS; mean (SD)]	25.2	(12.6)	19.2	(11.0)	<0.001	(0.509)
Difference absolute [mm VAS; mean (SD)]	−41.7	(15.8)	−47.7	(15.5)	<0.001	(0.380)
Relative [percent; mean (SD)]	−62.1	(16.3)	−71.4	(14.1)	<0.001	(0.605)
Within-cohort difference (significance (effect size))	<0.001	(2.804)	<0.001	(3.304)		
Quality-of-life—physical dimension at baseline [VR12-PCS; mean (SD)]	37.1	(8.3)	37.1	(8.3)	0.949	(0.003)
At end of week 4 [mean (SD)]	43.4	(8.5)	44.3	(8.5)	0.033	(0.103)
Difference absolute [mean (SD)]	6.3	(1.6)	7.1	(1.3)	<0.001	(0.570)
Relative [percent; mean (SD)]	17.9	(6.3)	20.3	(6.3)	<0.001	(0.382)
Within-cohort difference (significance (effect size))	<0.001	(0.754)	<0.001	(0.851)		
Quality-of-life—mental dimension at baseline [VR12-MCS; mean (SD)]	44.0	(8.3)	44.1	(8.5)	0.853	(0.009)
At end of week 4 [mean (SD)]	46.1	(8.3)	46.4	(8.6)	0.457	(0.036)
Difference absolute [mean (SD)]	2.1	(0.8)	2.3	(0.8)	<0.001	(0.284)
Relative [percent; mean (SD)]	4.9	(2.2)	5.4	(2.1)	<0.001	(0.229)
Within-cohort difference (significance (effect size))	<0.001	(0.247)	<0.001	(0.269)		
Overall wellbeing at baseline [MQHHF, NRS35; mean (SD)]	15.7	(5.8)	15.7	(5.9)	0.867	(0.008)
At end of week 4 [NRS35; mean (SD)]	28.2	(3.8)	29.8	(3.0)	<0.001	(0.474)
Difference absolute [mean (SD)]	12.5	(5.0)	14.1	(5.0)	<0.001	(0.317)
Relative [percent; mean (SD)]	64.6	(15.9)	73.2	(12.8)	<0.001	(0.593)
Within-cohort difference (significance (effect size))	<0.001	(2.537)	<0.001	(2.988)		
Quality-of-life impairment by pain at baseline [QLIP; mean (SD)]	18.9	(4.2)	18.9	(3.9)	0.881	(0.007)
At end of week 4 [mm VAS; mean (SD)]	31.6	(3.7)	33.3	(2.9)	<0.001	(0.489)
Difference absolute [mm VAS; mean (SD)]	12.8	(4.0)	14.4	(3.6)	<0.001	(0.415)
Relative [percent; mean (SD)]	60.6	(15.0)	68.3	(12.0)	<0.001	(0.564)
Within-cohort difference [significance (effect size)]	<0.001	(3.272)	<0.001	(4.183)		

Abbreviations: *n*: Number; %: percent; QUI: quinine; PRI: pridinol; mPDI: modified pain disability index; mm: millimeter; VAS: visual analog scale; SD: standard deviation; mPDI#6: subitem number 6 of the mPDI; VR12: Veterans Rand quality-of-life questionnaire, short form 12; PCS/MCS: physical and mental component scores of the VR12; MQHHF: Marburg questionnaire on habitual health findings; QLIP: quality-of-life impairment by pain inventory.

## Data Availability

The original data are not publicly available and access to individual-level data is restricted due to legal and ethical considerations. Requests to access the datasets should be directed to the corresponding author.
